# Socioeconomic characteristics, tobacco use, and service utilization by county poverty status among Oklahoma tobacco helpline registrants: A cross**-**sectional study

**DOI:** 10.18332/tpc/218374

**Published:** 2026-07-16

**Authors:** Gaurav Kumar, Laura A. Beebe, Motolani E. Ogunsanya, Mark P. Doescher, Ryan Nipp, Nirmal Choradia, Laili K. Boozary, Nathalia M. Machado, Darla E. Kendzor

**Affiliations:** 1 TSET Health Promotion Research Center, Stephenson Cancer Center, The University of Oklahoma Health CampusOklahoma CityUnited States; 2 Department of Biostatistics and EpidemiologyHudson College of Public Health, The University of Oklahoma Health CampusOklahoma CityUnited States; 3 Department of Family and Preventive MedicineThe University of Oklahoma Health CampusOklahoma CityUnited States; 4 Department of Internal Medicine, Section of Hematology/OncologyStephenson Cancer Center, The University of Oklahoma Health CampusOklahoma CityUnited States

**Keywords:** tobacco use, poverty, smoking cessation, tobacco helpline, persistent poverty

## Abstract

**Introduction:**

Residents of counties with higher poverty levels experience elevated smoking rates and disproportionate tobacco-related cancer mortality. This study examined differences in sociodemographic characteristics, tobacco use, and service utilization among Oklahoma Tobacco Helpline (OTH) registrants across county poverty designations.

**Methods:**

In this cross-sectional study, data were analyzed from 33847 adults registered with the OTH between 1 July 2023 and 30 June 2024. Counties were classified as persistent poverty (PPC: ≥20% of residents in poverty for ≥30 years), current poverty (CPC: ≥20% of residents in poverty currently for <30 years), or non-poverty (NPC: <20% of residents in poverty). Group differences were examined using the chi-squared test and analysis of variance. Adjusted multinomial logistic regression analyses evaluated associations between poverty status, tobacco use, and service utilization, controlling for age, sex/gender, race, education level, household income, health insurance, and chronic conditions.

**Results:**

Registrants residing in PPCs and CPCs differed from those in NPCs in terms of age and race, and they had lower education, income, and greater Medicaid coverage (all P<0.05). In adjusted models, PPC registrants had higher odds of smoking >1 pack/day (heavy smoking) (AOR=1.49; 95% CI: 1.30–1.70; p<0.001) and learning about OTH through personal networks (AOR=1.42; 95% CI: 1.16–1.73; p<0.001) compared with NPCs. Compared with registrants residing in non-poverty counties (NPCs), CPC registrants had higher odds of smoking >1 pack/day (AOR=1.39; 95% CI: 1.16–1.65; p<0.001) and learning about OTH through community/social organizations (AOR=1.77; 95% CI: 1.33–2.35; p<0.001). Service type and nicotine replacement therapy receipt/duration were similar across poverty designations.

**Conclusions:**

PPC and CPC registrants showed greater socioeconomic disadvantage and nicotine dependence than NPC registrants but had distinct information pathways. These findings highlight geographically concentrated poverty as a potential contributor to tobacco-related disparities and suggest that interventions addressing social and environmental conditions in PPCs may help enhance cessation engagement and reduce tobacco-related inequities.

## Introduction

Tobacco use, particularly cigarette smoking, is associated with increased risk for multiple cancers and contributes to worse cancer outcomes overall^1,2^. Cigarette smoking accounts for nearly 30% of all cancer-related deaths in the United States^3^ and is a major contributor to tobacco-related cancers, including lung, head and neck, bladder, pancreatic, and colorectal cancers. Lung cancer remains the leading cause of cancer-related death, with 80–90% of lung cancer deaths attributable to cigarette smoking^4,5^, surpassing the combined mortality rates of breast, colorectal, and prostate cancers. Although lung cancer affects all socio-economic groups, those with lower socio-economic status (SES) bear a disproportionately high burden, regardless of race or ethnicity^6^. Counties with persistent poverty (PPCs), where at least 20% of the population has lived in poverty for over 30 years, show elevated cancer mortality rates, including lung cancer and other tobacco-related cancers^7,8^. However, poverty is not uniform across counties, and comparing persistent poverty counties with counties experiencing current poverty or lower poverty levels can help determine whether long-term structural disadvantage is associated with distinct tobacco use patterns and cessation service engagement^9-11^.

PPCs are predominantly located in rural areas and have higher smoking prevalence rates than non-poverty counties (NPCs)^12,13^. In Oklahoma, over one-third of the population resides in rural areas^14^. Notably, Oklahoma has the eighth highest poverty rate in the US (15.7% vs 11.5% nationally)^15^. According to the Congressional Research Service, 15 of the 77 counties in Oklahoma met the criteria for persistent poverty in 2023^16^ (Supplementary file). Nearly all these counties are non-metropolitan, as classified by rural–urban continuum codes (RUCC) of 4–9^17^. Estimates from 2021 suggest that the prevalence of smoking in Oklahoma PPCs ranged from 19–30% (median=24%), significantly exceeding the state (18%) and national (15%) smoking rates^18^ at the same time. Similarly, the age-adjusted cancer mortality rate (based on the US standard) in Oklahoma PPCs was 204.5 per 100000 people^19^, 42% higher than the national rate (144.2)^20^ and 17% higher than the overall state rate (175.1)^19^. These alarmingly high smoking and cancer mortality rates highlight the urgent need for focused tobacco control and cancer prevention efforts in Oklahoma PPCs.

Although the Oklahoma Tobacco Helpline (OTH) offers statewide tobacco cessation services, including counseling and pharmacotherapy (e.g. nicotine replacement therapy), little is known about whether OTH registrant characteristics, tobacco use patterns, service utilization, and communication pathways (i.e. how individuals learn about the helpline) vary by county-level poverty status. Thus, this study sought to compare OTH registrants from persistent poverty counties, current poverty counties, and non-poverty counties in terms of sociodemographic characteristics, behavioral health, chronic disease, tobacco use, OTH service utilization, and communication pathways to Helpline awareness. These findings will help to identify potentially actionable information and guide targeted efforts to reduce tobacco-related cancer disparities in PPCs.

## Methods

### Data Source: Oklahoma Tobacco Helpline (OTH)

This cross-sectional study used data from individuals registered with the OTH during fiscal year 2024 (1 July 2023 – 30 June 2024). OTH provides free statewide tobacco cessation services, including phone coaching, digital tools, and nicotine replacement therapy (NRT). Participant registration data were self-reported and provided by RVO Health (formerly Optum), the OTH service provider. The registration dataset includes demographic information, tobacco use characteristics, and service utilization details. The setting for this study was statewide, including tobacco users who registered for services in all 77 Oklahoma counties. Eligible participants included adults aged ≥18 years who registered for OTH services during the study period and resided in Oklahoma. Registrants were excluded if county residence information was missing or invalid, or if registration data were incomplete for key analytic variables. This study involved data analysis of de-identified OTH data and was determined to be exempt from human subjects review by the University of Oklahoma Health Campus Institutional Review Board.

### Variables

#### County poverty designations

County poverty designation was assigned to each registrant based on the county of residence and defined according to the Congressional Research Service (CRS) persistent poverty classification^16^, which is based on U.S. Census Bureau American Community Survey (ACS) county-level poverty estimates^15^. The county poverty status was operationalized as follows: 1) PPCs included those with at least 20% of the population living in poverty for 30 years or more; 2) CPCs had a current poverty rate of 20% or more for less than 30 years (reflecting recent or emerging economic disadvantages); and 3) NPCs had poverty rates below 20%. A total of 15 Oklahoma counties met the criteria for PPCs, 10 were classified as CPCs, and 52 were classified as NPCs. The complete list of counties by poverty designation is provided in the Supplementary file.

#### Sociodemographic characteristics

Sociodemographic variables included age, sex/gender (female, male, gender minority [transgender female, transgender male, non-binary, genderqueer], refused), ethnicity (Hispanic, non-Hispanic, not known/refused), race (American Indian or Alaska Native, Black or African American, White, other/multiple races, not known/refused), education level (lower than high school, high school/GED, some college/technical training, college/technical degree, not known/refused), annual household income ($) (<20000, 20000–34999, 35000–74999, >75000, not known/refused), and health insurance (Medicaid, Medicare, military/veterans, private insurance, uninsured, not known/refused).

#### Behavioral and chronic health conditions

Participants self-reported behavioral health conditions, such as attention-deficit/hyperactivity disorder, anxiety, bipolar disorder, depression, substance use disorder (drug or alcohol abuse), gambling addiction, post-traumatic stress disorder, or schizophrenia (responses were combined and coded as yes [any behavioral health condition], no [no behavioral health conditions], or not known/refused). Participants also self-reported having any chronic health condition, including angina or chest pain, asthma, cancer, chronic bronchitis, diabetes (type 1, type 2, or prediabetes), heart attack, rapid or irregular heartbeat, heart failure, or stroke (responses were combined and coded as yes or no [any vs no health conditions]).

#### Tobacco use characteristics

Tobacco use variables included current cigarette use (self-reported smoking within the past 30 days at registration; yes, no), cigarettes smoked per day (<1, 1, >1 pack), minutes after waking to first tobacco use (≤5, >5), current e-cigarette use (yes, no), and current multiple tobacco product use (yes, no).

#### OTH service utilization and program participation

Service utilization variables included service type (coaching only, coaching + nicotine replacement therapy [NRT], NRT only, neither coaching nor NRT), number of NRT weeks sent (none, 2, 4–6, ≥8), number of coaching sessions completed (0, 1, ≥2), intervention intensity (levels of engagement in smoking cessation services: 0–2 [low], ≥3 [high]), and program type (behavioral health, pregnancy and postpartum, standard care coach+, standard care coach). Tobacco users were eligible for the behavioral health program if they reported one or more behavioral health conditions and responded ‘yes’ when asked if the condition interfered with quitting. The participants were eligible for up to seven coaching sessions and 12 weeks of NRT. Women who were pregnant, planning pregnancy, breastfeeding, or postpartum were eligible for up to seven coaching sessions and 8 weeks of NRT. Insurance status determined eligibility for either a standard care coach or coach + services. Coach + was available to most tobacco users and included five coaching sessions and up to eight weeks of mono or combination NRT. Those with private insurance were eligible for the standard care coach, which included five sessions and two weeks of mono NRT.

#### Sources of helpline information

Sources of OTH information (i.e. how registrants learned about OTH) included traditional media (e.g. commercials on local TV stations or radio shows), digital media (e.g. Facebook ads, online health websites), public/outdoor media (e.g. transit ads on buses, posters at public events), health campaigns/health services (e.g. CDC’s Tips campaign, local health services such as OK 211), personal networks (family members, friends, or healthcare providers), others (community or social service organizations, institutions, or programs), and not known/refused.

### Data analysis

Descriptive statistics, including means and standard deviations for continuous variables and frequencies and percentages for categorical variables, were calculated to describe participant characteristics. Chi-squared tests were used to examine associations between county poverty designations and categorical variables, while analysis of variance (ANOVA) was used to compare continuous variables across poverty categories. For variables showing significant differences by county poverty status, post-hoc tests were conducted to explore specific group differences. Pairwise comparisons were performed using Tukey’s HSD test for continuous variables and chi-squared *post hoc* tests for categorical variables to identify significant differences. Bonferroni adjustments were applied to control for multiple comparisons and to reduce the risk of Type I errors. Multinomial logistic regression models were evaluated after adjusting for age, sex, race, education level, household income, health insurance, and chronic conditions. Covariates were chosen using unadjusted group comparisons and conceptual considerations to account for key sociodemographic differences across county poverty groups, while minimizing overadjustment by excluding potential mediators. In preliminary models including behavioral conditions, e-cigarette use, number of coaching sessions, and intervention intensity, variance inflation factors (VIFs) exceeded 5 for several predictors, indicating multicollinearity^21^. Therefore, these variables were excluded from the final multinomial logistic regression models, in which all VIFs were <5. Missing data were minimal (≤5% across variables), and cases with missing values were handled using listwise deletion. All analyses were conducted using IBM SPSS Statistics, Version 31, with a two-tailed significance set at p<0.05.

## Results

### Participant characteristics

The sample included 33847 participants, divided by county poverty status as follows: PPCs (n=2915; 8.6%), CPCs (n=1653; 4.9%), and NPCs (n=29279; 86.5%). Overall, the mean age of participants was 47 years, most participants were female (60.1%), non-Hispanic (94.4%), and White (72.4%), with smaller proportions identified as American Indian or Alaskan Native (9.8%) and Black or African American (6.7%). Nearly half (45.7%) reported an annual household income below $20000, 30.4% had Medicaid insurance coverage, and 21.8% were uninsured. See Table 1 for participant characteristics according to county poverty status.

**Table 1:** Sociodemographic characteristics of adults registered with the Oklahoma Tobacco Helpline, by county poverty designation, cross-sectional study, 1 July 2023 – 30 June 2024 (N=33847)

Characteristics	Total n (%)	Persistent poverty n (%) (a)	Current poverty n (%) (b)	Non-poverty n (%) (c)	p
**Total**, n	33847	2915	1653	29279	
**Age** (years), mean (SD)	47.32 (14.9)	**46.71 (15.0)** ^b^	**48.21 (14.8)^a^**	47.33 (14.9)	**0.005**
**Sex/gender**					**<0.001**
Female	20358 (60.1)	**1897 (65.1)^c^**	1032 (62.4)	**17429 (59.5)^a^**	
Male	13189 (39.0)	**1006 (34.5)^c^**	609 (36.8)	**11574 (39.5)^a^**	
Gender minority	190 (0.6)	**6 (0.2)^c^**	6 (0.4)	**178 (0.6)^a^**	
Refused	109 (0.3)	6 (0.2)	6 (0.4)	97 (0.3)	
**Ethnicity**					0.390
Hispanic	1458 (4.3)	129 (4.4)	56 (3.4)	1273 (4.3)	
Non-Hispanic	31937 (94.4)	2750 (94.3)	1578 (95.5)	27609 (94.3)	
Not known/refused	451 (1.3)	36 (1.2)	19 (1.1)	396 (1.4)	
**Race**					**<0.001**
American Indian/Alaska Native	3329 (9.8)	**465 (16.0)** ^**b**^ ^**,** c^	**212 (12.8)** ^**a**^ ^**,**^ ^**c**^	**2652 (9.1)** ^**a**^ ^**,** b^	
Black/African	2262 (6.7)	**92 (3.2)** ^**b**^ ^**,** c^	**32 (1.9)** ^**a**^ ^**,**^ ^**c**^	**2138 (7.3)** ^**a**^ ^**,** b^	
White	24495 (72.4)	**2026 (69.5)** ^**b**^ ^**,** c^	**1264 (76.5)** ^**a**^ ^**,**^ ^**c**^	**21205 (72.4)** ^**a**^ ^**,** b^	
Other/multiple races	2961 (8.7)	**271 (9.3)** ^b^	**117 (7.1)** ^**a**^ ^**,**^ ^**c**^	**2573 (8.8)** ^b^	
Not known/refused	799 (2.4)	61 (2.1)	28 (1.7)	710 (2.4)	
**Education level**					**<0.001**
Lower than high school	4562 (14.5)	**465 (17.3)^c^**	**274 (17.8)^c^**	**3823 (14.1)** ^**a**^ ^**,** b^	
High school/GED	11871 (37.8)	1048 (39.0)	595 (38.6)	10228 (37.6)	
Some college/Technical training	8277 (26.4)	**653 (24.3)^c^**	399 (25.9)	**7225 (26.6)^a^**	
College/Technical degree	6269 (20.0)	495 (18.4)	**251 (16.3)^c^**	**5523 (20.3)** ^b^	
Not known/refused	429 (1.4)	29 (1.1)	23 (1.5)	377 (1.4)	
**Household income** ($)					**<0.001**
<20000	14333 (45.7)	**1432 (53.4)^c^**	**818 (53.2)^c^**	**12083 (44.5)** ^**a**^ ^**,** b^	
20000–34999	6317 (20.1)	517 (19.3)	297 (19.3)	5503 (20.3)	
35000–74999	5202 (16.6)	**350 (13.1)^c^**	**186 (12.1)^c^**	**4666 (17.2)** ^**a**^ ^**,** b^	
35000–74999	1630 (5.2)	**76 (2.8)^c^**	**54 (3.5)^c^**	**1500 (5.5)** ^**a**^ ^**,** b^	
>75000 Not known/refused	3883 (12.4)	305 (11.4)	184 (12.0)	3394 (12.5)	
**Health insurance**					**<0.001**
Medicaid	10302 (30.4)	**1028 (35.3)^c^**	536 (32.4)	**8738 (29.8)^a^**	
Medicare	5570 (16.5)	500 (17.2)	**317 (19.2)^c^**	**4753 (16.2)** ^b^	
Military/Veterans	590 (1.7)	**43 (1.5)** ^b^	**8 (0.5)** ^**a**^ ^**,** c^	**539 (1.8)** ^b^	
Private insurance	8863 (26.2)	**602 (20.7)^c^**	**377 (22.8)^c^**	**7884 (26.9)** ^**a**^ ^**,** b^	
Uninsured	7387 (21.8)	653 (22.4)	355 (21.5)	6379 (21.8)	
Not known/refused	1135 (3.4)	89 (3.1)	60 (3.6)	986 (3.4)	
**Behavioral health condition(s**)					0.05
Yes	18217 (53.8)	**1643 (56.4)^c^**	876 (53.0)	**15698 (53.6)^a^**	
No	14275 (42.2)	**1151 (39.5)** ^**b**^ ^**,** c^	**715 (43.3)^a^**	**12409 (42.4)^a^**	
Not known/refused	1354 (4.0)	121 (4.2)	62 (3.8)	1171 (4.0)	
**Chronic condition(s**)					**<0.001**
Yes	13762 (41.3)	**1198 (41.7)** ^b^	**791 (48.6)^a^^,^** ^**c**^	**11773 (40.9)** ^b^	
No	19539 (58.7)	**1674 (58.3)^b^**	**835 (51.4)^a^^,c^**	**17030 (59.1)^b^**	

Gender minority: transgender female, transgender male, non-binary, genderqueer. Superscripts (a, b, c): superscripts are placed next to the bold values to indicate which groups they differ from.

a Differs significantly from column a (persistent poverty counties)

b Differs significantly from column b (current poverty counties)

c Differs significantly from column c (non-poverty counties)

### Sociodemographic and health characteristics by county poverty status

In the descriptive analyses, most demographic characteristics differed significantly by county poverty status (p<0.05) (Table 1). *Post hoc* tests showed that PPC registrants were slightly younger than NPC registrants (46.7 vs 47.3 years). Registrants residing in PPCs had a higher proportion of females than those in NPCs (65.1% vs 59.5%), and fewer gender minority registrants were observed in PPCs (0.2% vs 0.6%). A higher proportion of American Indian or Alaska Native registrants were living in PPCs than in NPCs (16.0% vs 9.1%). Conversely, among registrants of NPCs, 7.3% were Black, which was higher than the proportion living in PPCs (3.2%) and CPCs (1.9%). Among registrants of CPCs, 76.5% were White, compared with 72.4% in NPCs and 69.5% in PPCs. No significant differences were found in the proportion of Hispanic registrants by county poverty status (p=0.39).

Socio-economic characteristics, including education level, income, and health insurance type, varied significantly by county poverty status (p<0.05) (Table 1). PPC (17.3%) and CPC (17.8%) registrants had higher proportions who reported lower than a high school education compared to NPCs (14.1%). A higher proportion of PPC (53.4%) and CPC (53.2%) registrants reported an annual income below $20000 compared with NPC registrants (44.5%). Medicaid insurance was more common in PPCs (35.3%) than in NPCs (29.8%), whereas private insurance was more common in NPCs (26.9%) than in CPCs (22.8%) and PPCs (20.7%).

Behavioral health conditions differed modestly across county poverty categories (p=0.05), while chronic conditions differed significantly (p<0.05), with CPC registrants reporting the highest prevalence (48.6%), followed by PPC registrants (41.7%) and NPC registrants (40.9%) (Table 1).

### Tobacco use by county poverty status

Tobacco use characteristics, including cigarette consumption, minutes to first tobacco use, e-cigarette use, and multiple tobacco product use, varied significantly by county poverty status (p<0.05) (Table 2). PPC (83.0%) and CPC (84.4%) registrants had higher smoking rates than NPC registrants (79.2%). Lighter smoking (<1 pack/day) was more common among the NPC registrants (44.6%) than among the PPC (40.1%) or CPC (36.8%) participants. In contrast, heavy smoking (>1 pack/day) was the highest among PPC (28.7%) and CPC (28.5%) registrants compared to NPCs (22.2%). Minutes to the first cigarette also varied, with PPC (57.9%) and CPC (59.7%) registrants more frequently reporting smoking within 5 min of waking than NPCs (54.4%). E-cigarette use was higher among PPC registrants (16.5%) than among NPCs (14.5%), and multiple tobacco product use was more common among PPCs (22.3%) than among NPCs (20.4%).

**Table 2:** Tobacco use characteristics, service utilization, and sources of helpline information among adults registered with the Oklahoma Tobacco Helpline, by county poverty designation, cross-sectional study, 1 July 2023 – 30 June 2024 (N=33847)

Characteristics	Total n (%)	Persistent poverty n (%) (a)	Current poverty n (%) (b)	Non-poverty n (%) (c)	p
**Total**, n	33847	2915	1653	29279	
**Cigarette use**					**<0.001**
Yes	27006 (79.8)	**2420 (83.0)** ^c^	**1395 (84.4)^c^**	**23191 (79.2)** ^**a**^ ^**,** b^	
No	6841 (20.2)	**495 (17.0)^c^**	**258 (15.6)^c^**	**6088 (20.8)^a^^,b^**	
**Cigarettes smoked per day** (pack)					**<0.001**
<1	11856 (43.8)	**972 (40.1)^c^**	**514 (36.8)^c^**	**10370(44.6)** ^**a**^ ^**,** b^	
1	8963 (33.1)	756 (31.2)	484 (34.7)	7723 (33.2)	
>1	6233 (23.1)	**694 (28.7)^c^**	**398 (28.5)^c^**	**5141 (22.2)^a^^,b^**	
**Minutes to first tobacco use after waking**					**<0.001**
≤5	18614 (55.0)	**1688 (57.9)^c^**	**987 (59.7)^c^**	**15939 (54.4)** ^**a**^ ^**,** b^	
>5	15233 (45.0)	**1227 (42.1)^c^**	**666 (40.3)^c^**	**13340 (45.6)^a^^,b^**	
**E-cigarette use**					**<0.001**
Yes	4985 (14.7)	**482 (16.5)^c^**	267 (16.2)	**4236 (14.5)^a^**	
No	28862 (85.3)	**2433 (83.5)^c^**	1386 (83.8)	**25043 (85.5)^a^**	
**Multiple tobacco product use**					**0.023**
Yes	6979 (20.6)	**651 (22.3)^c^**	366 (22.1)	**5962 (20.4)^a^**	
No	26868 (79.4)	**2264 (77.7)^c^**	1287 (77.9)	**23317 (79.6)^a^**	
**Services type**					**0.006**
Coaching only	1417 (4.2)	137 (4.7)	80 (4.8)	1200 (4.1)	
Coaching+NRT	20046 (59.2)	**1750 (60.0)** ^b^	**916 (55.4)** ^**a**^ ^**,** c^	**17380 (59.4)** ^b^	
NRT only	10317 (30.5)	834 (28.6)	528 (31.9)	8955 (30.6)	
Neither coaching nor NRT	2067 (6.1)	194 (6.7)	**129 (7.8)^c^**	**1744 (6.0)^b^**	
**Weeks of NRT sent by helpline**					**0.035**
None	3484 (10.3)	331 (11.4)	**209 (12.6)^c^**	2944 (10.3)^b^	
2	3274 (9.7)	**232 (8.0)^c^**	145 (8.8)	**2897 (9.9)^a^**	
4–6	20641 (61.0)	1785 (61.2)	1006 (60.9)	17850 (61.0)	
≥8	6448 (19.1)	567 (19.5)	293 (17.7)	5588 (19.1)	
**Number of coaching sessions completed**					**0.031**
0	12384 (36.6)	**1028 (35.3)** ^b^	**657 (39.7)** ^**a**^ ^**,** c^	**10699 (36.5)** ^b^	
1	12871 (38.0)	**1128 (38.7)** ^b^	**580 (35.1)** ^**a**^ ^**,** c^	**11163 (38.1)** ^b^	
≥2	8592 (25.4)	759 (26.0)	416 (25.2)	7417 (25.3)	
**Intervention intensity**					**0.031**
0–2	14683 (43.4)	**1225 (42.0)** ^b^	**761 (46.0)^a^**	12697 (43.4)	
3–4	19164 (56.6)	**1690 (58.0)^b^**	**892 (54.0)^a^**	16582 (56.6)	
**Program type**					**0.009**
Behavioral health	12464 (36.8)	1088 (37.3)	620 (37.5)	10756(36.7)	
Pregnancy and postpartum	1089 (3.2)	112 (3.8)	52 (3.1)	925 (3.2)	
Standard care coach+	13946 (41.2)	**1286 (44.1)^c^**	702 (42.5)	**11958 (40.8)^a^**	
Standard care coach	6348 (18.8)	**429 (14.7)^c^**	279 (16.9)	**5640 (19.3)^a^^,^^b^**	
**Sources of helpline information**					**0.001**
Traditional media (e.g. TV, news, radio)	5308 (18.4)	**395 (16.5)^c^**	**219 (15.8)^c^**	**4694 (18.8)** ^**a**^ ^**,** b^	
Digital media (e.g. social media, online)	1682 (5.8)	**148 (6.2)** ^b^	**125 (9.0)** ^**a**^ ^**,** c^	**1409 (5.6)** ^b^	
Public/outdoor media (e.g. transit ad)	480 (1.7)	30 (1.3)	**13 (0.9)^c^**	**437 (1.7)** ^b^	
Health campaigns/health services (e.g. OK 211, CDC Tips campaign)	14839 (51.5)	1227 (51.3)	706 (51.0)	12906 (51.6)	
Personal networks (e.g. family/friends, healthcare providers)	3478 (12.1)	291 (12.2)	156 (11.3)	3031 (12.1)	
Other (community/social service organization /institution/program)	2333 (8.1)	**253 (10.6)^c^**	126 (9.1)	**1954 (7.8)^a^**	
Not known/refused	674 (2.3)	50 (2.1)	39 (2.8)	585 (2.3)	

NRT: nicotine replacement therapy. Superscripts (a, b, c): superscripts are placed next to the bold values to indicate which groups they differ from.

a Differs significantly from column a (persistent poverty counties)

b Differs significantly from column b (current poverty counties)

c Differs significantly from column c (non-poverty counties)

### Oklahoma Tobacco Helpline service utilization by county poverty status

OTH service utilization, including service combinations (coaching and NRT), NRT receipt, and program enrollment, differed significantly by county poverty status (p<0.05) (Table 2). PPC (60.0%) and NPC (59.4%) registrants were more likely to receive coaching + NRT than CPCs (55.4%), whereas CPCs were most likely to receive neither coaching nor NRT (7.8% vs 6.0% in NPCs). CPC registrants were also less likely to receive NRT from the OTH (12.6%) than NPCs registrants (10.3%). NPCs were more likely to receive NRT for two weeks than PPCs (9.9% vs 8.0%, respectively). Most registrants (61.0%) accessed 4–6 weeks of NRT, which did not vary by county type. CPC registrants more frequently completed no coaching sessions (39.7%) than PPCs (35.3%) and NPCs (36.5%). Further, PPC (38.7%) and NPC (38.1%) registrants were more likely to complete one coaching session than CPCs (35.1%). PPC registrants (58.0%) were also more likely to engage in higher intensity interventions (≥3 sessions) than were CPCs (54.0%), whereas NPC engagement (56.6%) was comparable to that of PPCs. Program enrollment patterns varied, with PPC registrants having the highest participation in the standard care coach + program (44.1%), followed by CPCs (42.5%) and NPCs (40.8%). In contrast, NPCs had higher participation in the standard care coach program (19.3%) than did PPCs (14.7%) and CPCs (16.9%). Behavioral health programs were most frequently used among PPC (37.3%) and CPC (37.5%) registrants, whereas pregnancy and postpartum program enrollment was slightly higher among PPCs (3.8%) than among CPCs (3.1%) and NPCs (3.2%).

### Sources of helpline information

Sources of helpline information for the OTH registrants varied according to county poverty status (p<0.05) (Table 2). Traditional media sources were cited more by NPC registrants (18.8%) than by PPCs (16.5%) or CPCs (15.8%). CPC registrants reported digital media sources more frequently (9.0%) than did PPCs (6.2%) and NPCs (5.6%). PPC registrants more frequently reported learning about OTH through community/social service organizations compared with NPC registrants (10.6% vs 7.8%).

### Multinomial logistic regression

The multinomial logistic regression model showed good overall fit and no multicollinearity issues. After adjusting for sociodemographic covariates (age, sex, race, education level, income, insurance, and chronic conditions), several differences emerged in tobacco use and information sources according to county poverty designation (p<0.05) (Table 3). Among participants who smoked cigarettes, registrants from PPCs had significantly higher odds of smoking >1 pack/day (heavy smoking) (AOR=1.49; 95% CI: 1.30–1.70; p<0.001) but did not differ significantly in the likelihood of smoking 1 pack/day (AOR=1.07; 95% CI: 0.97–1.24) compared with smoking <1 pack/day than those from NPCs. No significant differences were observed in the minutes to first tobacco use, multiple product use, service type received, program type, or number of weeks of NRT supplied. However, registrants from PPCs were more likely to learn about OTH through personal networks compared with traditional media (reference group) (AOR=1.42; 95% CI: 1.16–1.73; p<0.001) compared with registrants from NPCs.

**Table 3:** Adjusted multinomial logistic regression of tobacco use, service utilization, and sources of helpline information, among adults registered with the Oklahoma Tobacco Helpline, by county poverty designation, cross-sectional study, 1 July 2023 – 30 June 2024 (N=33847)

Variables	Persistent poverty (vs NPC) AOR (95% CI)	Current poverty (vs NPC) AOR (95% CI)
**Tobacco use characteristics**		
**Cigarette use**		
Yes	3.29 (0.44–24.21)	1.71 (0.23–12.59)
No (ref.)	1	1
**Cigarette packs smoked per day**		
>1	1.49 (1.30–1.70)**	1.39 (1.16–1.65)**
1	1.07 (0.97–1.24)	1.22 (1.04–1.43)*
<1 (ref.)	1	1
**Minutes to first use tobacco after waking**		
≤5	1.00 (0.89–1.12)	0.06 (0.92–1.22)
>5 (ref.)	1	1
**Multiple tobacco product use**		
Yes	1.10 (0.97–1.24)	1.11 (0.94–1.31)
No (ref.)	1	1
**Service utilization**		
**Service type**		
Coaching only	0.95 (0.69–1.32)	0.93 (0.64–1.35)
Coaching+NRT	0.95 (0.67–1.34)	0.71 (0.48–1.07)
NRT only	0.96 (0.68–1.35)	0.91 (0.61–1.37)
Neither coaching nor NRT (ref.)	1	1
**Weeks of NRT supplied**		
≥8	0.91 (0.67–1.23)	0.84 (0.58–1.22)
4–6	0.91 (0.69–1.21)	0.88 (0.63–1.23)
2^§^	-	-
None (ref.)	1	1
**Program type**		
Behavioral health	0.98 (0.86–1.11)	1.00 (0.85–1.17)
Pregnancy and postpartum	1.11 (0.81–1.51)	0.84 (0.52–1.34)
Standard care coach	1.15 (0.85–1.56)	1.09 (0.76–1.58)
Standard care coach+ (ref.)	1	1
**Sources of helpline information**		
Other (community/social organization)	1.26 (0.99–1.61)	1.77 (1.33–2.35)**
Digital media	0.74 (0.45–1.22)	0.53 (0.24–1.15)
Health campaigns/services	1.06 (0.92–1.22)	1.13 (0.94–1.36)
Public/outdoor media	1.00 (0.82–1.22)	1.01 (0.78–1.31)
Personal networks	1.42 (1.16–1.73)**	1.23 (0.93–1.63)
Traditional media (ref.)	1	1

Multinomial logistic regression models were adjusted for sociodemographic variables, including age, sex, race, education level, household income, health insurance status, and chronic conditions.Reference group: registrants from non-poverty counties. NRT: nicotine replacement therapy. § Non-estimable parameters due to redundancy or zero cell counts; SPSS automatically sets these coefficients to zero. Significance: *p<0.05; **p<0.001.

AOR: adjusted odds ratio.

Similarly, registrants from CPCs were more likely to smoke > 1 pack/day (AOR=1.39; 95% CI: 1.16–1.65; p<0.001) and 1 pack/day (AOR=1.22; 95% CI: 1.04–1.43; p=0.013) versus smoking <1 pack/day (reference) compared with registrants from NPC. No significant differences were observed in minutes to first tobacco use, multiple product use, service type received, program type, or the number of weeks of NRT supplied. Registrants from CPCs were more likely to learn about OTH through community or social organizations compared with traditional media (AOR=1.77; 95% CI: 1.33–2.35; p<0.001) compared with NPCs.

## Discussion

This study examined differences in sociodemographic characteristics, tobacco use, service utilization characteristics, and OTH registrants’ sources of helpline information of OTH registrants by county poverty status, given prior research demonstrating higher tobacco use and cancer mortality in PPCs^7,12^. Overall, descriptive findings indicated that PPC registrants were younger and more likely to report American Indian/Alaska Native race and female sex, and more frequently had Medicaid insurance coverage compared with registrants from NPCs. PPC and CPC registrants were also more socio-economically disadvantaged, with lower levels of education and lower household incomes than NPC registrants. Tobacco patterns also differed; PPC and CPC registrants were more likely than NPC registrants to smoke cigarettes, engage in heavier smoking, report greater nicotine dependence, use e-cigarettes, and use multiple tobacco products. Although service utilization was largely comparable across poverty categories after adjusting for covariates, pathways to learning about the Helpline differed, with PPC and CPC registrants gaining information more often through personal or community networks than through traditional or digital media. Together, these findings point to structural inequities in tobacco burden and communication access for residents of PPCs and CPCs and can guide OTH outreach efforts to better engage poverty-affected communities and reduce tobacco-related disparities.

Although OTH services are available across Oklahoma, no prior studies have assessed whether service utilization differs by poverty status or whether PPC-specific factors influence helpline reach and engagement. In this sample, PPC registrants comprised less than one-tenth of all OTH users, which is consistent with the proportion of Oklahoma residents living in PPCs (8.2%) (Supplementary file Table). However, PPC residents had greater socio-economic disadvantage, such as lower level of education, lower income, and higher Medicaid enrollment, which are well-documented correlates of smoking and predictors of persistent nicotine dependence^22,23^. Importantly, PPCs show concentrated and multiple intersecting forms of disadvantage, including economic hardship, rurality, and racialized inequities which may collectively amplify tobacco-related risk. These socio-economic patterns align with prior work showing that groups overrepresented in PPCs (e.g. American Indians and females) experience disproportionately high tobacco-related morbidity and lower cessation rates^24,25^. To date, however, few studies have explicitly examined county poverty status as a distinct structural determinant, and additional research is needed to confirm whether persistent poverty independently amplifies tobacco-related disparities. Nonetheless, our findings indicate that PPC and CPC registrants showed heavier smoking and greater use of multiple tobacco products, behaviors linked to lower cessation success and worse long-term outcomes.

Prior research has demonstrated that cessation is difficult for socio-economically disadvantaged populations due to mixed motivation to quit, more positive expectations about smoking, smoking as a social norm, lower self-efficacy, and financial constraints that limit access to pharmacotherapy and sufficient treatment intensity^26,27^. These challenges may be especially pronounced in PPCs and CPCs, in which chronic stress, limited access to healthcare resources, and environments that do not promote good health can reinforce tobacco use^22,26^. In the descriptive analyses, the PPC and CPC registrants were more likely than NPC registrants to smoke within 5 min of waking, use e-cigarettes, and smoke > 1 pack/day. After adjusting for sociodemographic and socio-economic factors, the PPC and CPC registrants were significantly more likely to be heavy smokers (>1 pack/day), and CPC registrants were also more likely to smoke one pack per day than less than one pack per day. After adjusting for individual characteristics, smoking intensity differed by county poverty designation. Although time to first cigarette and multiple product use did not differ significantly by county poverty status in the adjusted models, the persistent pattern of heavier smoking in PPCs and CPCs may reflect the cumulative effects of chronic stress, reduced healthcare access, and fewer cessation resources in poverty areas^26^.

This study also provides insights into OTH service utilization by county poverty status. In descriptive analyses, CPC registrants were somewhat more likely to receive no NRT or complete no coaching sessions, while PPC and NPC registrants were more likely to receive coaching plus NRT and complete at least one coaching session. However, these differences were not significant in the adjusted multinomial models, suggesting that once individuals enroll in OTH, key cessation services (coaching and NRT) are delivered relatively equally across counties. This indicates that variability in service utilization is driven more by individual sociodemographic factors that cluster within high-poverty counties, rather than by county poverty status alone. The ability to provide equal access to evidence-based cessation support regardless of geographical poverty level is a key strength of OTH^28^. Given that the PPC and CPC registrants presented with higher nicotine dependence and more complex tobacco use patterns, it is plausible that they may require more intensive or tailored cessation support (e.g. extended NRT, more coaching sessions, or integrated behavioral health counseling)^10,29^.

Perhaps one of the most salient findings of this study relates to how the registrants learned about OTH. In both descriptive and adjusted analyses, PPC and CPC registrants were more likely to report non-media than traditional media sources. PPC registrants had higher odds of learning about the Helpline through personal networks (family, friends, and healthcare providers) than traditional media, whereas CPC registrants had higher odds of learning through community or social service organizations. NPC registrants, in contrast, more often cited traditional media as their source of information. These findings highlight that statewide digital and mass media campaigns alone may be insufficient in high-poverty counties and underline the importance of implementation strategies that leverage clinics, tribal health systems, community-based organizations, social service agencies, and community health workers as critical pathways for Helpline dissemination and policy-relevant outreach in PPCs and CPCs^30,31^.

Our findings also highlight the unique treatment needs of registrants of PPCs and CPCs. Within our sample of Oklahoma Tobacco Helpline registrants, a higher proportion of American Indian/Alaska Native individuals and females resided in PPCs compared with NPCs. Therefore, interventions that consider culture, race, and sex/gender may be beneficial. Culturally relevant approaches for American Indian and Alaska Native communities, such as involving tribal leadership in program design, integrating traditional values, addressing historical and contemporary trauma, and embedding Helpline referrals within tribal health clinic workflows may enhance effectiveness^32-34^. For women, tailored counseling and pharmacotherapy that address caregiving responsibilities, stress, and co-occurring mental health concerns may improve outcomes^33^. The higher reliance on Medicaid among PPC registrants further underlines the importance of policies that strengthen Medicaid coverage of cessation medications and counseling, reduce administrative barriers, and support proactive outreach to Medicaid enrollers^35^.

Comparisons between PPCs and CPCs suggest a continuum of disadvantages. On many indicators, PPC and CPC registrants were more similar to each other than to NPC registrants, including higher smoking prevalence, heavier smoking, greater multiple tobacco product use, and higher behavioral health burden. However, CPC registrants had the highest prevalence of chronic conditions and appeared less engaged in coaching or NRT in descriptive findings, whereas PPC registrants were more likely to complete higher intensity interventions and rely on personal networks for helpline information. Previous research suggests that individuals experiencing poverty may face practical barriers such as intermittent phone service, limited minutes or data, irregular work schedules, transportation challenges, or lower digital literacy^36^. Future qualitative or mixed-methods studies could clarify these barriers and inform strategies to improve engagement and retention among CPC registrants.

### Strengths and limitations

This study had several notable strengths. The large sample size of >33000 participants strengthened the reliability and generalizability of our findings. The use of statewide, real-world OTH registration and service utilization data strengthens the implementation relevance and ecological validity of the results for population-level tobacco control efforts. Another key strength is the classification of counties into PPCs, CPCs, and NPCs, which provides valuable insights into the influence of county poverty status on the characteristics and engagement of OTH registrants to inform efforts to increase outreach and engagement. This study examined the similarities and differences between PPCs and CPCs, both of which showed an elevated risk of tobacco-related health problems.

This study also has several limitations. Smoking cessation outcomes were not examined by county poverty status because of limited statistical power, underscoring the need for long-term follow-up data. Findings may have limited generalizability beyond Oklahoma given the state’s unique sociodemographic, resource context and because the sample included only individuals who registered for Oklahoma Tobacco Helpline services. The cross-sectional design precludes causal inference between poverty status, tobacco use, and helpline engagement. Because county poverty designation was assigned at the county level, analyses did not incorporate clustering adjustments at the county level, and unmodeled within-county correlation may have influenced variance estimates. Although models adjusted for key sociodemographic factors, residual confounding may remain due to unmeasured variables such as neighborhood deprivation, access to healthcare, local tobacco policies, or psychosocial stressors^22,26^. County-level poverty measures may also not fully reflect individual-level socio-economic circumstances, which could lead to under- or over-estimation of associations between poverty designation and tobacco-related outcomes^9^. Tobacco use measures were self-reported and may be subject to recall or social desirability bias. Further, county-level poverty classifications may overrepresent rural poverty while overlooking disadvantaged urban populations; smaller geographical units (e.g. ZIP codes or census tracts) may better capture these disparities. Additionally, because, OTH services are delivered remotely, unmeasured rural barriers including poor connectivity, limited broadband access, lower digital literacy, and fewer local resources may have influenced engagement. Finally, several variables (e-cigarette use, coaching sessions, and intervention intensity) were excluded from the adjusted multinomial logistic regression models due to multicollinearity and should be explored in future studies.

## Conclusions

Study findings indicated differences in tobacco use, nicotine dependence, and cessation service engagement across counties with varying poverty levels. Registrants from the PPCs and CPCs shared socio-economic challenges but showed distinct service patterns. These findings emphasize the need for cessation strategies tailored to higher poverty communities, such as strengthening outreach through trusted community and healthcare partners, improving access to counseling and pharmacotherapy, and addressing structural barriers including limited healthcare availability and resource constraints to reduce tobacco and cancer disparities in PPCs and CPCs. Future research should focus on developing focused cessation interventions for populations living in disadvantaged areas to reduce tobacco use and cancer disparities.

## Supplementary material

Supplementary file Material 1. Descriptives of Oklahoma Counties
