# Supplementary material for: Socioeconomic characteristics, tobacco use, and service utilization by county poverty status among Oklahoma tobacco helpline registrants: A cross-sectional study
Source: Tob Prev Cessat. 2026 Jul 16;12:10.18332/tpc/218374. doi: 10.18332/tpc/218374 (PMC13386057; doi:10.18332/tpc/218374)
Supplement: Supplementary file Material 1. — Descriptives of Oklahoma Counties [file TPC-12-41-s001.pdf]

Supplemental Table. Descriptives of Oklahoma Counties.

Category (2021)[1]	County	Population (2023)[2]	RUCC Code (2023)[3]
Persistent Poverty	ADAIR	19,627	8
Persistent Poverty	CADDO	26,214	6
Persistent Poverty	CHEROKEE	48,185	6
Persistent Poverty	GREER	5,466	9
Persistent Poverty	HARMON	2,392	9
Persistent Poverty	HASKELL	11,832	8
Persistent Poverty	HUGHES	13,436	9
Persistent Poverty	JEFFERSON	5,347	8
Persistent Poverty	LATIMER	9,526	9
Persistent Poverty	MCCURTAIN	15,970	7
Persistent Poverty	OKFUSKEE	11,300	8
Persistent Poverty	PAYNE	83,352	4
Persistent Poverty	SEMINOLE	23,565	7
Persistent Poverty	SEQUOYAH	40,291	3
Persistent Poverty	TILLMAN	6,869	8
Poverty	BECKHAM	22,042	7
Poverty	CHOCTAW	14,276	9
Poverty	COAL	5,266	6
Poverty	COTTON	5,427	3
Poverty	JOHNSTON	10,216	9
Poverty	KIOWA	8,398	8
Poverty	LE FLORE	49,596	6
Poverty	MCINTOSH	39,889	8
Poverty	OTTAWA	30,287	6
Poverty	TEXAS	20,371	7
Non Poverty	ALFALFA	5,673	8
Non Poverty	ATOKA	14,525	9
Non Poverty	BEAVER	5,018	9
Non Poverty	BLAINE	8,539	8
Non Poverty	BRYAN	48,967	6
Non Poverty	CANADIAN	175,829	1
Non Poverty	CARTER	48,596	5
Non Poverty	CIMARRON	2,191	9
Non Poverty	CLEVELAND	301,193	1
Non Poverty	COMANCHE	121,574	3
Non Poverty	CRAIG	14,494	6
Non Poverty	CREEK	73,332	1
Non-Poverty	CUSTER	41,703	5
Non Poverty	DELAWARE	4,286	6
Non Poverty	DEWEY	3,648	9
Non Poverty	ELLIS	62,023	9
Non Poverty	GARFIELD	25,865	3
Non Poverty	GARVIN	57,375	6
Non Poverty	GRADY	4,083	1
Non Poverty	GRANT	3,190	1
Non Poverty	HARPER	24,669	9
Non Poverty	JACKSON	43,641	8

Non Poverty	KAY	15,481	4
Non Poverty	KINGFISHER	34,562	8
Non Poverty	LINCOLN	53,029	1
Non Poverty	LOGAN	10,296	1
Non Poverty	LOVE	28,266	9
Non-Poverty	MAJOR	47,072	8
Non-Poverty	MARSHALL	30,660	9
Non-Poverty	MAYES	19,603	6
Non-Poverty	MCCLAIN	7,581	1
Non-Poverty	MURRAY	13,754	9
Non-Poverty	MUSKOGEE	66,677	4
Non-Poverty	NOBLE	10,832	8
Non-Poverty	NOWATA	9,438	8
Non-Poverty	OKLAHOMA	808,866	1
Non-Poverty	OKMULGEE	37,035	1
Non-Poverty	OSAGE	46,130	1
Non-Poverty	PAWNEE	15,864	1
Non-Poverty	PITTSBURG	43,479	7
Non-Poverty	PONTOTOC	38,396	7
Non-Poverty	POTTAWATOMIE	73,791	4
Non-Poverty	PUSHMATAHA	10,800	9
Non-Poverty	ROGER MILLS	3,295	9
Non-Poverty	ROGERS	100,248	1
Non-Poverty	STEPHENS	44,014	4
Non-Poverty	TULSA	682,868	1
Non-Poverty	WAGONER	89,280	1
Non-Poverty	WASHINGTON	53,706	4
Non-Poverty	WASHITA	10,736	9
Non-Poverty	WOODS	8,564	7
Non-Poverty	WOODWARD	19,947	7

Note: The RUCC (Rural-Urban Continuum Codes) classification is based on the USDA (U.S Department of Agriculture) ERS (Economic Research Service) 2023 designations, which categorize counties based on population size and proximity to metro areas.

RUCC Code Descriptions:
1 - Metro, population of 1 million or more
3 - Metro, population of fewer than 250,000
4 - Nonmetro, urban population of 20,000 or more, adjacent to a metro area
5 - Nonmetro, urban population of 20,000 or more, not adjacent to a metro area
6 - Nonmetro, urban population of 5,000 to 19,999, adjacent to a metro area
7 - Nonmetro, urban population of 5,000 to 19,999, not adjacent to a metro area
8 - Nonmetro, urban population of fewer than 5,000, adjacent to a metro area
9 - Nonmetro, urban population of fewer than 5,000, not adjacent to a metro area

[1] *Dalaker, J. The 10-20-30 provision: defining persistent poverty counties (2023). Congressional Research Service, (Report No. R45100). Retrieved from https://crsreports.congress.gov/product/pdf/R/R45100. Accessed on February 18, 2025.*

[2] *U.S. Department of Agriculture, Economic Research Service. (2024). 2023 Rural-Urban Continuum Codes. Retrieved from https://www.ers.usda.gov/data-products/rural-urban-continuum-codes. Accessed on February 18, 2025.*

[3] *U.S. Census Bureau. (2024). Annual Estimates of the Resident Population for Counties in Oklahoma: April 1, 2020, to July 1, 2023 [Data set]. Retrieved from https://www2.census.gov/programs-surveys/popest/tables/2020-2023/counties/totals/co-est2023-chg-40.xlsx. Accessed on February 18, 2025.*

© 2026 Kumar G. et al.
